# Severe form of Ebstein’s anomaly associated with ventricular septal defect

**DOI:** 10.1186/s44215-023-00039-0

**Published:** 2023-04-18

**Authors:** Yuji Fuchigami, Takaya Hoashi, Shigeki Yoshiba, Kentaro Hotoda, Haruhiro Nagase, Yukino Iijima, Takaaki Suzuki

**Affiliations:** 1 Departments of Pediatric Cardiac Surgery, Saitama Medical University International Medical Center, Hidaka, Saitama Japan; 2 Pediatric Cardiology, Saitama Medical University International Medical Center, Hidaka, Saitama Japan

**Keywords:** Ebstein’s anomaly, Cone tricuspid valve repair, Bidirectional Glenn

## Abstract

Although the ventricular septal defect (VSD) that coexists with the severe form of Ebstein’s anomaly was thought to be beneficial for hemodynamics, it is unclear whether biventricular repair is always possible. Ebstein’s anomaly with VSD was diagnosed at 4 days of age based on a heart murmur and mild cyanosis. The cardiothoracic ratio was 78% and the Celermajer index was 1.6 (grade 4), but the hemodynamics were stable and tricuspid valve regurgitation was less than mild. Pulmonary overcirculation developed, and therefore, biventricular repair was attempted at 4 months of age, consisting of VSD patch closure, atrial septal defect partial closure, and the cone reconstruction of the tricuspid valve. Due to persistent cyanosis, however, nitric oxide inhalation and high flow nasal oxygen inhalation could not be discontinued. Catheter examination showed a cardiac index of 2.1 L/min/m^2^, moderate tricuspid regurgitation, and a pulmonary-to-systemic blood flow ratio of 0.64, and thus, takedown to one and one-half ventricular circulation using bidirectional superior cavopulmonary anastomosis, atrial communication closure, and right pulmonary artery banding was performed. The patient was discharged on postoperative day 25. Four months after discharge, the patient is doing well with home oxygen therapy.

## Background

Coexistence of a ventricular septal defect (VSD) with Ebstein’s anomaly with hypoplastic functional right ventricle (FRV) was thought to be beneficial for hemodynamics [1]. However, when surgical intervention is required, it is unclear whether biventricular circulation can always be established [2–4]. Herein, we report a patient with a severe form of Ebstein’s anomaly with coexisting VSD for whom biventricular repair using the cone reconstruction of the tricuspid valve was attempted at 4 months of age. This resulted in takedown to one and one-half ventricular repair with additional bidirectional superior cavopulmonary anastomosis 1 month later.

## Case presentation

### Case

**Fig. 1:**
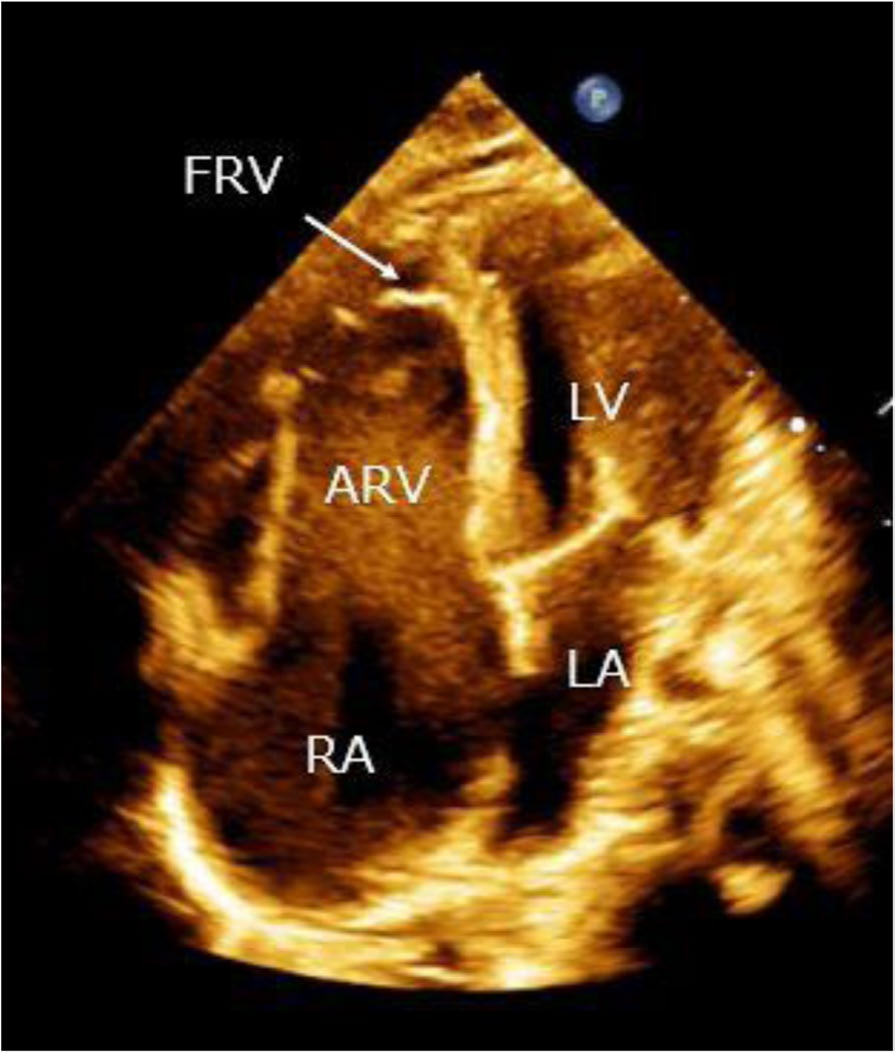
An apical four-chamber view of trans-thoracic echocardiography. RA, right atrium; LA, left atrium; ARV, atrialized right ventricle; FRV, functional right ventricle

An infant girl was born at 38 weeks gestation by normal vaginal delivery, and she weighed 2382 g. At age 4 days, a heart murmur and cyanosis were noticed, and she was transferred to the regional hospital. Ebstein’s anomaly, atrial septal defect (ASD), and VSD were diagnosed. Although the ductus arteriosus had already closed, her homodynamic condition was stable with a small dose of oral diuretics. Because pulmonary overcirculation had gradually developed, she was transferred to our hospital at 4 months of age. Upon arrival, an echocardiogram showed hypoplastic FRV (Fig. 1). The Carpentier classification was type C and the Celermajer index was 1.6 (grade 4). The septal leaflet displacement was 16.8 mm (70 mm/m^2^). A VSD 4 mm in diameter was located at the perimembranous portion with trabecular extension. Antegrade pulmonary blood flow was mainly from the left ventricle through the VSD, and interestingly, tricuspid valve regurgitation was less than mild. Preoperative cardiac catheter examination showed excessive pulmonary overcirculation, but pulmonary vascular resistance was not increased (Table 1).

**Table 1:** Pre- and postoperative cardiac catheter examinations results

	Pre-operative data	Postoperative data
Qp/Qs	3.50	0.64
Qp (L/min/m^2^)	7.38	2.39
Qs (L/min/m^2^)	2.11	3.70
Rp/Rs	0.13	0.25
Rp (units × m^2^)	1.63	1.89
Rs (units × m^2^)	12.33	7.56
RVP (Sys/-EDP mmHg)	34/ − 8	13/ − 7
PAP (Sys/Dias (Mean) mmHg)	30/12 (20)	13/8 (11)
TR (sellers grade)	I	III
Respiratory condition	Room air	FiO2 0.85, NO 10 ppm

*Qp/Qs* Pulmonary to systemic blood flow ratio, *Rp/Rs* Pulmonary to systemic vascular resistance ratio, *RVP* Right ventricular pressure, *Sys* Systolic blood pressure, *Dias* Diastolic blood pressure, *EDP* End diastolic pressure, *PAP* Pulmonary artery pressure, *Mean* Mean blood pressure, *TR* Tricuspid regurgitation, *FiO2* Fraction of inspired oxygen, *NO* Nitric oxide

**Fig. 2:**
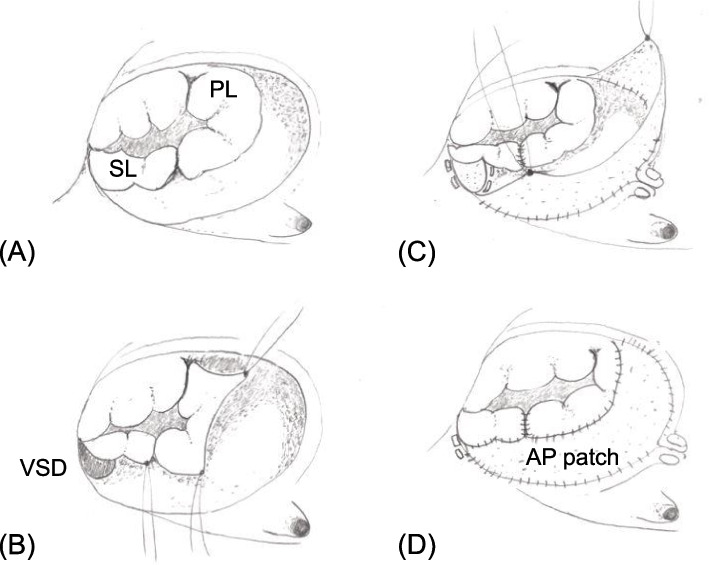
The septal leaflet (SL) and posterior leaflet (PL) of tricuspid valve severely displaced towards the apex (**A**). The SL and PL were detached from the annulus (**B**). The detached SL and PL were whole circumferentially augmented by the fresh autopericardial patch (AP) (**C**), then it was anastomosed to the true annulus (**D**)

Operation consisted of the cone reconstruction of the tricuspid valve, VSD closure, and partial ASD closure. The septal and posterior leaflets of tricuspid valve were severely displaced toward the apex and adhered to the underlying myocardium (Fig. 2A). After VSD patch closure, both septal and posterior leaflets were detached from the annulus and underlying myocardial surface, and attempts were made to create a cone-shaped unicusp (Fig. 2B). However, the leaflets were too small to make a complete cone shaped valve. Thus, the detached septal and posterior leaflets were whole circumferentially augmented by the fresh autopericardial patch. The width of the patch was an about 30 mm (Fig. 2C), which was then anastomosed to the true annulus (Fig. 2D). A 15-mm bouqie was passed through the reconstructed tricuspid valve orifice. The atrialized right ventricle (RV) was not plicated not to reduce the right ventricular volume. The ASD was closed using a with ePTFE patch with a 4-mm fenestration.

The patient was extubated on post-operative day 1. Since then, respiratory failure and cyanosis remained, and nitric oxide and high flow nasal oxygen inhalation could not be discontinued. Trans-tricuspid peak flow velocity after biventricular repair was 1.2 m/s, tricuspid regurgitation was moderate, and right-to-left shunting through the atrial communication was recorded using transthoracic echocardiography. Tadalafil, selexipag, and macitentan were administered without any improvement in the clinical condition. Cardiac catheter examination at post-operative day 40 showed that the pulmonary-to-systemic blood flow ratio was reduced to 0.64. The cardiac index was 2.1 L/min/m^2^. Therefore, the takedown to one and one-half ventricular circulation by bidirectional superior cavopulmonary anastomosis with right pulmonary artery banding was performed. At that time, atrial communication was completely closed. Postoperative course was stable, and she was discharged on post-operative day 25. Transthoracic echocardiogram at discharge showed no tricuspid stenosis and moderate tricuspid regurgitation, and the peak tricuspid regurgitation pressure gradient was 20 mmHg. Four months after discharge, the patient is doing well with home oxygen therapy.

## Discussion

Patients with Ebstein`s anomaly and hypoplastic FRV due to severely displaced septal and posterior tricuspid valve leaflets toward the RV apex show significant cyanosis and low cardiac output syndrome just after birth. Therefore, single ventricular palliation is required during the neonatal period. Coexisting VSD is thought to be beneficial for such a hemodynamic instability for several reasons [1]. First, blood flow through the VSD goes directly to the pulmonary artery instead of antegrade pulmonary blood flow from the hypoplastic FRV, which would mitigate cyanosis without leaving the duct open. Second, blood flow through the VSD to the FRV would generate both pressure and volume overloads to the RV outlet, which may promote RV growth. Both patients in a previous report survived beyond 1 year of age without any intervention, regardless of their disease severity [1]. Moreover, patient 2 was doing well after her VSD was spontaneously closed.

When surgery is required, all of the reported patients with Ebstein’s anomaly and VSD whose detailed clinical course was described and who underwent biventricular repair survived (Table 2) [2–4]. None of the patients experienced takedown to one and one-half ventricular circulation thereafter. Our patient ultimately required takedown to one and one-half ventricular circulation. She showed significant postoperative tricuspid regurgitation, although her preoperative regurgitation was minimal. Additionally, the atrialized RV was not plicated at the initial surgery because atrialized RV plication would make the apparent RV cavity too small to establish biventricular circulation. If tricuspid regurgitation can be effectively regulated and noncontractile and if obstructive (for left ventricle) atrialized RV is plicated, biventricular circulation might be established. Otherwise, VSD closure without any tricuspid valve procedure might be the other option to mimic patient 2 from a previous report [1].

On the contrary, the severity of Ebstein’s anomaly was more significant in the presented case (Table 2). Both the Great Ormond Street Echocardiography score and the Celermajer index were grade 4. Therefore, significant postoperative cyanosis and tricuspid regurgitation should be derived from the RV dysfunction. Although coexisting VSD plays a hemodynamically favorable role in Ebstein’s anomaly, a previous study on patients with neonatally diagnosed Ebstein’s anomaly or tricuspid dysplagia included two deaths in patients with VSD [5]. The detailed clinical situation was not described; but such a result would justify our management because single ventricular palliation could be avoided, and the patient was doing well. Of course, long-term follow up is required to detect late complications after one and one-half ventricular repair, such as superior vena cava syndrome, pulmonary arteriovenous malformations, and venous aneurysm formation [6].

**Table 2:** Previously reported patient cases with Ebstein’s anomaly and ventricular septal defect who underwent biventricular repair

Case	Age at Op	Carpentier classification	TR	GOSE score	Celermajer Index grade	Procedure (TV procedure)	Outcome
1	23 days	C	Moderate	2	2	BVR (Cone)	Alive
2	3 months	C	Severe	3	1	BVR (Cone)	Alive Tracheostomy
3	20 days	N/A	Severe	3	3	BVR (Danielson)	Alive
4	< 3 months	A	Trivial	N/A	N/A	BVR (N/A)*	Alive
5	< 3 months	B	Severe	N/A	N/A	BVR (N/A)*	Alive
6	< 3 months	B	Severe	N/A	N/A	BVR (N/A)*	Alive

*Op* Operation, *TR* Tricuspid regurgitation, *GOSE score* GREAT Ormond Street Echocardiography score, *TV* Tricuspid valve, *BVR* Biventricular repair, *N/A* Not available

## Conclusion

Unique and stable hemodynamics could be maintained during the neonatal and early infantile periods when VSD coexists with a severe form of Ebstein’s anomaly. Biventricular repair with the cone reconstruction of the tricuspid valve was attempted at 4 months of age, which resulted in takedown to one and one-half ventricular repair 1 month later.

## Data Availability

There are no additional data to disclose.

## Abbreviations

FRV: Functional right ventricle
VSD: Ventricular septal defect
RV: Right ventricle
